# Cooperative binding of AP-1 and TEAD4 modulates the balance between vascular smooth muscle and hemogenic cell fate

**DOI:** 10.1242/dev.139857

**Published:** 2016-12-01

**Authors:** Nadine Obier, Pierre Cauchy, Salam A. Assi, Jane Gilmour, Michael Lie-A-Ling, Monika Lichtinger, Maarten Hoogenkamp, Laura Noailles, Peter N. Cockerill, Georges Lacaud, Valerie Kouskoff, Constanze Bonifer

**Affiliations:** 1 Institute of Biomedical Research, College of Medicine and Dentistry, University of Birmingham, Birmingham B15 2TT, UK; 2 CRUK Manchester Institute, University of Manchester, Manchester M20 4BX, UK

**Keywords:** AP-1, TEAD4, Hippo signalling, Hematopoietic specification, ESC differentiation, Signalling hubs

## Abstract

The transmission of extracellular signals into the nucleus involves inducible transcription factors, but how different signalling pathways act in a cell type-specific fashion is poorly understood. Here, we studied the regulatory role of the AP-1 transcription factor family in blood development using embryonic stem cell differentiation coupled with genome-wide transcription factor binding and gene expression analyses. AP-1 factors respond to MAP kinase signalling and comprise dimers of FOS, ATF and JUN proteins. To examine genes regulated by AP-1 and to examine how it interacts with other inducible transcription factors, we abrogated its global DNA-binding activity using a dominant-negative FOS peptide. We show that FOS and JUN bind to and activate a specific set of vascular genes and that AP-1 inhibition shifts the balance between smooth muscle and hematopoietic differentiation towards blood. Furthermore, AP-1 is required for *de novo* binding of TEAD4, a transcription factor connected to Hippo signalling. Our bottom-up approach demonstrates that AP-1- and TEAD4-associated cis-regulatory elements form hubs for multiple signalling-responsive transcription factors and define the cistrome that regulates vascular and hematopoietic development by extrinsic signals.

## Abstract (precis)

**Highlighted article:** AP-1 activates the expression of vascular genes in hemogenic endothelial cells and is required for *de novo* binding of the Hippo signaling transcription factor TEAD4.

## INTRODUCTION

The hematopoietic system has been a long-standing model for general principles driving the transcriptional control of cell fate decisions. During vertebrate embryonic development, the emergence of definitive hematopoietic stem cells (HSCs) occurs in the dorsal aorta (Medvinsky and Dzierzak, 1996), where cells of a specialized hemogenic endothelium (HE) undergo an endothelial-to-hematopoietic transition (EHT), lose adherence and, as newborn HSCs, move to other sites of the embryo (Boisset et al., 2011; Eilken et al., 2009; Kissa and Herbomel, 2010; Lancrin et al., 2009). Each of these developmental transitions is regulated by an orchestrated interplay of stage-specific transcription factors (TFs). For example, SCL/TAL1 is required for the differentiation of HB cells to HE cells (D'Souza et al., 2005; Lancrin et al., 2009), while RUNX1 is essential for the EHT (Chen et al., 2009; Lancrin et al., 2009). These TFs are connected to common and distinct target genes within a dynamic transcriptional network (Goode et al., 2016). However, although the roles of transcriptional regulators of hematopoietic differentiation are beginning to be understood, it is less clear how outside signals direct their activity and drive developmental stage-specific gene expression.

The transmission of signals into the nucleus involves surface molecules, such as receptor kinases, and inducible TFs at the receiving end. Most inducible transcription factors are expressed in multiple cell types and cooperate with tissue-restricted or other inducible factors by binding to their cognate cis-regulatory elements and altering gene expression in a signalling-dependent way. How this process is coordinated and connected to the signalling network is poorly understood and difficult to study, because signalling pathways are highly dynamic, consisting of a myriad of different components that operate in a cell type-specific fashion and displaying multiple types of crosstalk. However, the sequences that hard-wire the response to signals into our DNA are the same in all cells. By identifying and studying the function of signalling-responsive cis-regulatory elements and their interacting factors, we are able to obtain a first insight into how signal transduction processes are coordinated at the genomic level.

A paradigm for inducible transcription factors is the activator protein 1 (AP-1) family of transcription factors, which are typical targets of MAP kinases, including ERK and JNK (Chang and Karin, 2001), that enhance their transcriptional activity through phosphorylation (Angel et al., 1987; Karin and Smeal, 1992). Generally, AP-1 factors promote gene expression, often in response to stimuli such as growth factors. The AP-1 family comprises FOS (FOS, FOSB, FOSL1, FOSL2) and JUN (JUN, JUNB, JUND) proteins, but also ATF (ATFa, ATF-2, ATF-3) and JDP (JUN dimerization proteins, JDP1, JDP2) proteins, all of which are structurally and functionally related and act as dimers (Hess et al., 2004; Jochum et al., 2001). While JUN family proteins can dimerize with FOS, JUN, ATF and JDP proteins, FOS family proteins can heterodimerize with only JUN family members. Several findings point to an important role of this TF family at early stages of hematopoietic specification: (1) work from our group found that AP-1 motifs were enriched in open chromatin regions and colocalized with TF-binding sites that were specific to HE cells differentiated from mouse embryonic stem cells (ESCs) (Goode et al., 2016; Lichtinger et al., 2012); (2) JUN knockout (KO) mice die around the onset of HSC emergence (Eferl et al., 1999); (3) AP-1 was reported to play a role in *Xenopus* hematopoiesis (Lee et al., 2012); (4) in zebrafish, the transcriptional co-repressor NCoR silences *Fos* transcription and NCoR knockdown leads to inhibition of HE formation (Wei et al., 2014); (5) AP-1 activation is involved in the stimulation of engraftment of HSCs by epoxyeicosatrienonic acids (Li et al., 2015); and (6) FOS has been identified as a crucial factor together with GATA2, GFI1B and ETV6, in the reprogramming of mouse embryonic fibroblasts (MEFs) to blood cells (Pereira et al., 2013). However, none of these studies has identified the global genomic targets responsible for these effects. In addition, the expression of individual AP-1 family members, and thus the dimer composition, varies depending on the cellular context. Owing to the redundancy in this system, the analysis of the general role of AP-1 factors has been difficult.

In this study, we gained a first insight into the role of the AP-1 factor family as a whole using *in vitro* differentiated mouse ESCs as a model. During ESC differentiation, the first blood cells derive from the hemangioblast (HB), a mesodermal cell type capable of differentiating into vascular smooth muscle (SM), endothelial and hematopoietic cells (Choi et al., 1998; Huber et al., 2004; Kennedy et al., 1997; Stefanska et al., 2014). We expressed a dominant-negative FOS (dnFOS) peptide from a doxycycline (DOX)-inducible promoter and thereby abolished all AP-1 DNA-binding activity (Olive et al., 1997). A surprising result of our work was the finding that global AP-1 inhibition, in spite of the near-ubiquitous expression of this factor family, is compatible with hematopoietic specification, whereby in differentiating hemangioblast cells FOS and JUN together bind to and activate a core set of vascular effector genes. Importantly, we found that at these genes AP-1 does not act alone but cooperates with TEAD4, a mediator of the Hippo signalling pathway (Meng et al., 2016), which we have previously shown to be essential for hematopoietic specification (Goode et al., 2016). AP-1 inhibition abolished TEAD4 binding at these genes, thus uncovering the mechanism of the interdependency of the two signalling pathways. Our data therefore show how inducible transcription factors are integrated at the genomic level to form signalling hubs that modulate the balance between cell fates.

## RESULTS

### Global AP-1 inhibition affects differentiation of ESCs via hemangioblast to hematopoietic cells

**Fig. 1.**
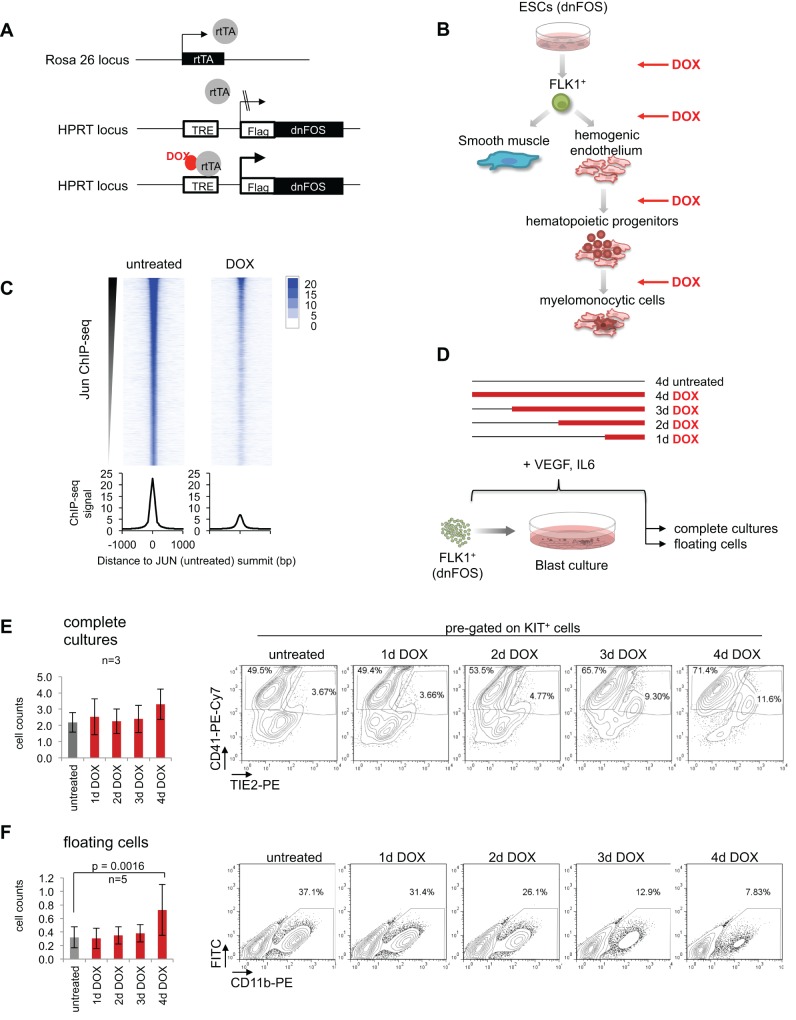
**AP-1 inhibition affects differentiation of ESCs to hemangioblast, to hemogenic and to hematopoietic cells.** (A) Schematic representation of the targeted HPRT-gene locus of the DOX-inducible dnFOS-expressing A17 2lox mouse ESC line (dnFOS ESCs). (B) Overview of the *in vitro* differentiation of ESCs to blood cells and the corresponding time course of dnFOS induction (DOX). (C) JUN ChIP-seq in uninduced and DOX-treated dnFOS cells. FLK1^+^ dnFOS cells were cultured under blast culture conditions for 1 day±1 µg/ml DOX. Cells of complete cultures were double crosslinked and chromatin was used for JUN ChIP followed by genome-wide sequencing. The JUN ChIP-seq signal in the untreated dataset and the corresponding signal detected in the DOX dataset are shown. (D) Schematic overview of the DOX-induction time course approach during blast culture. FLK1^+^ dnFOS cells were cultured for 4 days under blast culture conditions. Cells were either left untreated (4 days untreated) or DOX was added from start of culture (for 4 days=4 days DOX), at day 1 (3 days DOX), day 2 (2 days DOX) or day 3 (1 day DOX) of culture. At day 4, complete cultures and floating cells were analysed for cell numbers and surface marker profiles by flow cytometry. (E) Total cell counts (left) and a representative CD41-/Tie2-specific flow cytometric analysis of pre-gated cKit^pos^ cells (right) of day 4 complete blast cultures that were either left untreated or DOX-treated for 1 day, 2 days, 3 days or 4 days (data are mean±s.d., *n*=3). (F) Total cell counts (left) and a representative CD11b-specific flow cytometric analysis (right) of day 4 floating cells derived from dnFOS blast cultures that were either left untreated or DOX treated for 1 day, 2 days, 3 days or 4 days (data are mean ±s.d., *n*=3).

The family of AP-1 transcription factors consists of diverse members, most of which can heterodimerize and are likely to compensate for each other's absence. Therefore, we inhibited all AP-1 activity directly and globally using a dominant-negative version of FOS (dnFOS). This peptide contains a dimerization domain for binding to JUN family proteins and an acidic extension for blocking the DNA-binding domain of JUN, thus preventing both the formation of JUN:JUN, JUN:ATF and JUN:FOS dimers, and their binding to DNA (Olive et al., 1997). In order to examine a role of AP-1 factors at different stages of hematopoietic specification, we employed *in vitro* ESC differentiation that has previously been used to recapitulate and investigate the different steps in hematopoietic specification (Fig. S1A) (Goode et al., 2016; Lancrin et al., 2010). We constructed an ESC line carrying a doxycycline (DOX)-inducible Flag-tagged dnFOS allele (Fig. 1A) (Kyba et al., 2002). We ensured that the peptide inhibited AP-1-driven gene activation in a luciferase assay (Fig. S1B), that its expression was tightly regulated (Fig. S1C) and that the protein was present in every cell (Fig. S1D), and demonstrated that induction of the construct indeed blocked JUN binding to DNA in a genome-wide fashion (Fig. 1C and Fig. S1E).

Several members of the AP-1 family are expressed at one or more stages of *in vitro* ESC differentiation into blood (Fig. S1F). To be able to study the molecular mechanism of AP-1 function in detail, we first had to examine at which stages AP-1 activity was crucially required. To this end, we induced dnFOS by addition of DOX at distinct time points of ESC differentiation.

As outlined in Fig. 1B (and Fig. S1A), ESCs were differentiated into embryoid bodies (EBs) and after 3.75 days HB cells were enriched by purifying cells positive for the VEGF receptor (FLK1^+^). Subsequently, FLK1^+^ cells were kept for up to 4 days in the presence of IL-6 and VEGF under blast culture (BC) conditions where they differentiated to vascular smooth muscle (SM) cells or successively to the following cell types: (1) early hemogenic endothelial cells (HE1, KIT^+^TIE2^+^CD41^−^), (2) late hemogenic endothelial cells fully committed to blood but still adherent (HE2, KIT^+^TIE2^+^CD41^+^) and (3) floating hematopoietic progenitor cells that have undergone the EHT (HP, KIT^+^TIE2^−^CD41^+^). The inhibition of AP-1 activity during the establishment of HB cells by adding DOX to EB cultures led to increases in total cell number within EB cultures and in the proportion of FLK1^+^ cells (Fig. S1G,H). To examine the role of AP-1 at later differentiation stages, we expressed dnFOS during BC by either inducing freshly purified FLK1^+^ cells (4 days of induction) or adding DOX subsequently at day 1, day 2 or day 3 of BC (Fig. 1D). After 4 days, complete cultures and floating cells (containing progenitor cells) were assessed for cell count and phenotypic composition, respectively. Despite no significant change in overall cell number (Fig. 1E, left panel), complete blast cultures contained a higher proportion of KIT^+^ cells (Fig. S1I,J) and HE2 cells after dnFOS induction (Fig. 1E, right panel; Fig. S1K). This effect was strongest when AP-1 was inhibited at the very beginning of blast culture. Moreover, we found that an early block of AP-1 (4 days DOX treatment) led to a significant increase in the amount of floating cells (Fig. 1F, left). However, myeloid commitment of such cells, as measured by CD11b surface marker expression, was significantly diminished (Fig. 1F, right panel; Fig. S1L). Moreover, when AP-1 was inhibited only transiently in the first 24 h of blast culture with subsequent DOX washout and culture medium replacement, numbers of floating cells at day 3 were increased to the same extent as observed after a continuous 3-day treatment period (Fig. S1M). By contrast, proportions of CD11b+ cells were decreased to a lesser extent when DOX was withdrawn after 1 day (Fig. S1N).

Together, these results suggest that AP-1 is involved in modulating the transitions of several distinct stages of blood development, supporting or impairing respective cell fates. However, in spite of the near-ubiquitous expression of this factor family, global AP-1 inhibition does not lead to gross disturbances in blood cell specification.

### Inhibition of AP-1 at the hemangioblast stage shifts the balance between vascular and blood cell development

**Fig. 2.**
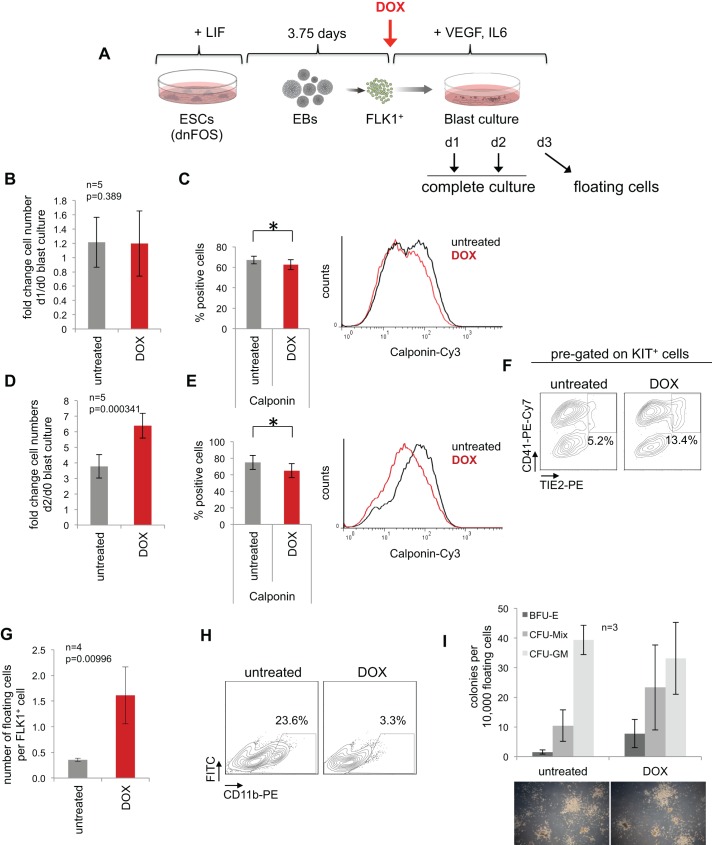
**AP-1 inhibition at the hemangioblast stage enhances cell proliferation and shifts the balance between vascular and blood cell development.** (A) Experimental setup: dnFOS ESCs were differentiated for 3.75 days as EBs. FLK1^+^ HB cells were purified and subsequently cultured±1 µg/ml DOX under blast culture conditions for 1 day, 2 days or 3 days before either complete cultures (day 1 and day 2) or floating cells (day 3) were analysed. (B) Fold changes of cell numbers at day 1 compared with day 0 (=seeded cell numbers) of complete dnFOS blast cultures with and without DOX induction (data are mean ±s.d., *n*=5, *t*-test). (C) Complete day 1 dnFOS blast cultures with and without DOX induction were assessed by intracellular SM cell-specific calponin staining (data are mean ±s.d., *n*=4, *t*-test, *P=0.019). A representative flow cytometric analysis and the summary of three experiments is shown (for other smooth muscle cell markers and statistical summary, see Fig. S2). (D) Fold changes of cell numbers at day 2 compared with day 0 (=seeded cell numbers) of complete dnFOS blast cultures with and without DOX induction (data are mean ±s.d., *n*=5, *t*-test). (E) Complete day 2 dnFOS blast cultures with and without DOX induction were assessed by intracellular SM cell-specific calponin staining. (data are mean ±s.d., *n*=3, *t*-test, *P=0.038). A representative flow cytometric analysis and the summary of three experiments is shown (for other smooth muscle cell markers and statistical summary, see Fig. S2). (F) The cell composition of complete day 2 dnFOS blast cultures ±DOX was analysed by flow cytometry using antibodies against KIT, TIE2 and CD41. A representative contour plot of pre-gated KIT^+^ cells is shown. (G) The number of dnFOS floating cells with and without DOX induction at day 3 per FLK1^+^ cell that was seeded at day 0 (data are mean±s.d., *n*=4). (H) Floating cells of day 3 dnFOS blast cultures with and without DOX induction were harvested and stained with a CD11b-specific antibody prior to flow cytometric analysis. Representative contour plots are shown. (I) Floating cells derived from day 3 dnFOS blast cultures with and without DOX induction were harvested and plated into methylcellulose medium for a hematopoietic colony assay (in the absence of DOX). After 10 days, colonies were counted and classified as BFU-E, CFU-Mix and CFU-GM (data are mean ±s.d., *n*=3). Examples of colonies are shown at the bottom.

Having shown that blocking all AP-1 activity is compatible with differentiation, we next examined whether this factor was involved in regulating cell fate. To this end, we studied the differentiation of HB cells by analysing complete blast cultures ±DOX after 1 and 2 days of induction, as well as floating cells after 3 days (Fig. 2A). Although cell numbers at day 1 were unaffected by AP-1 inhibition, we measured a significant reduction in the proportion of SM cells as assessed by intracellular flow cytometry using three different SM cell markers: SMA, SM22α and calponin (Fig. 2B,C and Fig. S2D). Endothelial marker expression was unaltered with the exception of a slight increase in FLK1^+^ cells in DOX-treated cultures (Fig. S2E). Further, when analysing day 2 blast cultures we observed significantly increased cell numbers (Fig. 2D), with overall no difference in apoptosis (Fig. S2A). DOX-induced cells showed higher S- and G2/M-cell cycle phase contribution and expressed significantly lower levels of the negative cell cycle regulator gene *Cdkn2a* (p16) (Fig. S2B,C), which explains the enhanced proliferation. As in the time course experiment (Fig. 1E), the frequency of HE2 cells was also significantly increased when AP-1 was inhibited for 2 days only (Fig. 2F, Fig. S2H), whereas SM cell proportions were significantly lower (Fig. 2E, Fig. S2F) and endothelial cells remained unaffected (Fig. S2G). These results demonstrate that the gain of hematopoietic HE2-type cells was paralleled by a loss of SM cells. The number of non-adherent, floating cells that had undergone the EHT after a 3-day culture and dnFOS-induction period was significantly increased by more than fourfold when compared with untreated cultures (Fig. 2G). We next characterized these cells by surface marker flow cytometry. The analysis revealed that the proportions of KIT^+^, CD41^+^ and CD71^+^ cells within floating cells emerging upon AP-1 inhibition were comparable with untreated cells, demonstrating that hematopoietic commitment had happened (Fig. S2I,J). However, although floating cells of untreated cultures showed expression of CD45 and CD11b, indicating their maturation and myeloid commitment, DOX-treated cells failed to express these markers and appeared to be blocked in differentiation (Fig. S2I,J and Fig. 2H). We further noticed a slight but significant increase in CD71^high^ cells upon AP-1 inhibition, which was reported to represent a population of primitive erythroblasts (Chao et al., 2015). To test whether floating cells derived from dnFOS-induced cultures have the potential to form normal colony when compared with untreated cultures, we performed colony-forming assays in the absence of DOX. Overall, we detected similar colony formation capacity, with similar numbers and sizes of colonies (Fig. 2I). Although the myeloid commitment in dnFOS-induced blast cultures was inhibited (Fig. 2H and Fig. S2I,J), progenitor cells derived from DOX-treated blast cultures were able to give rise to myeloid cell-containing CFU-GM- and CFU-Mix-type colonies when DOX was withdrawn, with a slight bias towards colonies with erythroid contribution (BFU-E and CFU-Mix). Together, these experiments demonstrate that AP-1 inhibition by dnFOS in differentiating HB cells shifts the balance between smooth muscle and hemogenic cell fates and reversibly blocks myeloid commitment, suggesting a dual role of AP-1 in this developmental pathway, promoting both smooth muscle and myeloid cell types.

### AP-1 inhibition does not alter global transcriptional networks but affects expression of key stage-specific genes

**Fig. 3.**
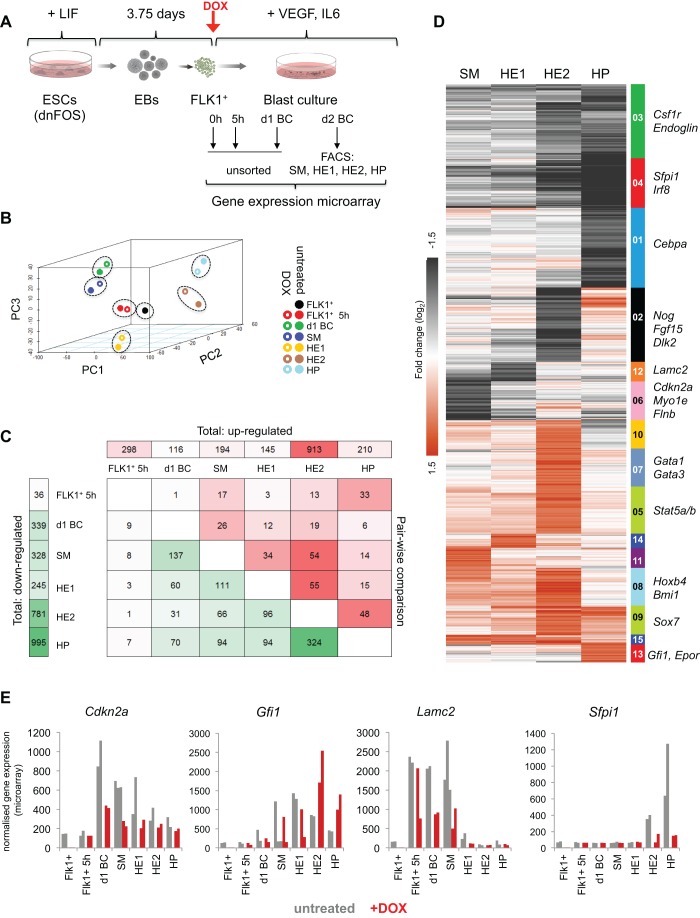
**dnFOS induction causes distinct changes to global gene expression programmes.** (A) Experimental setup: dnFOS ESCs were differentiated for 3.75 days as EBs. FLK1^+^ HB cells were purified and subsequently cultured with and without 1 µg/ml DOX under blast culture conditions for 5 h, 24 h or 48 h. Freshly purified FLK1^+^ cells (=0 h), 5 h and 1 day cultures with and without DOX induction were used directly for RNA extraction, whereas day 2 cultures with and without DOX induction were sorted by FACS into pure populations of SM, HE1, HE2 and HP cells prior to RNA extraction. RNA was used for genome-wide gene expression arrays. Each population was analysed in duplicate. (B) Principle component analysis with three components for all 13 indicated cell types. (C) Pairwise comparison of microarray data from untreated and DOX-treated cells. The numbers of significantly (at least twofold) up- and downregulated genes are shown. The numbers at the top indicate the genes that are upregulated (DOX versus untreated samples) for each time point, the numbers on the left represent genes that are downregulated (DOX versus untreated samples) for each time point. The numbers within the table show how many of the up-/downregulated genes at each time point are also mis-regulated (green, down; red, up) at other time points. (D) SM, HE1, HE2 and HP samples with and without DOX induction were used for k-means clustering by fold change of genes that change expression at least twofold upon dnFOS induction. For the resulting 15 clusters of genes, a heatmap was generated and some genes of interest are indicated next to their respective cluster. (E) Expression values of exemplary genes over the course of differentiation with and without DOX induction based on data from microarrays. Individual replicates for each sample are depicted.

To gain insight into how AP-1 regulates cell fate, we focused on the differentiation of HB cells into SM and HE cells. We investigated the transcriptional changes induced by blocking AP-1 activity using microarray gene expression analysis with RNA extracted from freshly purified FLK1^+^ HB cells, complete blast cultures after 5 h and 1 day of induction, as well as from FACS-sorted SM, HE1, HE2 and HP cells at day 2 of blast culture (Fig. 3A, Fig. S3A,B). Principal component analysis revealed that samples of the same cell type clustered closely together with or without dnFOS induction, indicating that AP-1 inhibition did not globally alter the gene expression patterns of induced cells (Fig. 3B, Fig. S3C). However, we identified a number of genes that were at least twofold differentially expressed (Fig. 3C, Fig. S3D and Table S1). Consistent with AP-1 generally functioning as an activator, we found more downregulated than upregulated genes. K-means clustering of differentially expressed genes in the sorted cell populations SM, HE1, HE2 and HP (Fig. 3D) established a total of 15 clusters, each containing genes with similar expression fold change patterns over the four cell types. We also evaluated their gene ontology (GO) terms (Table S2 and Fig. S3E). Clusters 3, 4 and 1 retrieved genes that were expressed at lower levels in HE2 and HP cells such as the myeloid regulators *Csf1r*, *Sfpi1* (*Pu.1*) and *Cebpa*, which was in line with the reduced number of CD11b^+^ cells observed in DOX-treated blast cultures. Genes upregulated in HP cells (cluster 13) were associated with erythrocyte differentiation and clusters 12 and 6 comprised genes that were downregulated in SM and HE1 cells, e.g. *Lamc2*, *Myo1e*, *Flnb*, *Serpine1* or *Wt1* (Fig. 3D,E and Fig. S3G). These gene products are associated with vascular cell types (GO terms Fig. S3E and Duim et al., 2015) and their decreased expression may reflect the lower contribution of vascular SM cells upon dnFOS-induction. We also found a group of HSC-related genes, including *Hoxb4*, *Bmi1* and *Gfi1*, that were expressed at higher levels in the absence of AP-1 activity (Fig. 3D,E, Fig. S3G, with manual validations shown in Fig. S3F). In SM and HE1 cells, blocking AP-1 activity affected vascular genes, whereas at later stages in HE2 and especially HP cells myeloid gene expression was reduced. In summary, despite their widespread expression and promiscuous involvement in multiple signalling processes, the absence of AP-1 during cell differentiation does not deregulate large sets of genes, but influences cell fate decisions by impacting on a limited set of key stage-specific regulator and effector genes.

### AP-1 is required for the transient activation of vascular genes in the hemogenic endothelium

**Fig. 4.**
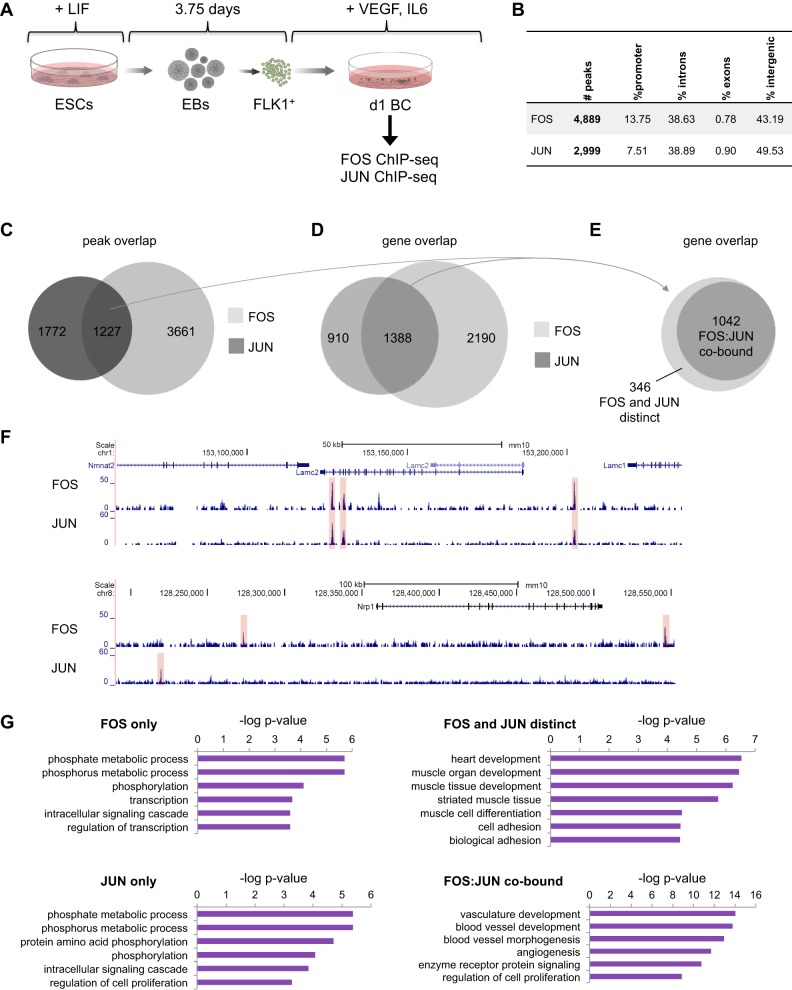
**During hemangioblast commitment, FOS and JUN bind to genes involved in blood vessel development, cell adhesion and cell signalling.** (A) Experimental setup: Bry-GFP WT ESCs (JUN) or dnFOS ESCs (FOS) were differentiated as indicated. FLK1^+^ cells were purified and cultured for 1 day under blast culture conditions in the absence of DOX. Cells of complete cultures were double crosslinked as described in the Materials and Methods, and chromatin was used for FOS and JUN ChIP followed by genome-wide sequencing. (B) Binding and genome localization statistics of FOS and JUN peaks within the mouse genome. (C) Overlap of peaks detected in FOS and JUN ChIP-seq datasets. (D) Overlap of genes associated with FOS and JUN peaks. (E) Overlap of genes associated with intersecting FOS and JUN peaks (C) and genes associated with FOS- and JUN-binding events (D). (F) Representative genome browser screen shots of FOS and JUN ChIP-seq at the *Lamc2* and *Nrp1* loci. (G) Gene ontology analysis of genes bound by FOS only, JUN only, FOS:JUN co-bound and bound by FOS and JUN distinctly. The top GO terms and the corresponding −log *P*-value are shown.

The most common AP-1 complex consists of FOS and JUN proteins. To identify targets for these two factors, we performed ChIP-seq in day 1 blast culture cells, which mostly comprise SM cells with the rest being HE1/2 cells (Fig. 4A, Fig. S4A). As AP-1 proteins only transiently bind to their templates (Biddie et al., 2011), we employed a double-crosslink procedure that greatly enhanced ChIP signals (see Materials and Methods). We found a total of 4889 and 2999 binding events for FOS- and JUN-binding sites, respectively, with the majority of them being at distal regulatory elements at either intronic or intergenic sites (Fig. 4B, Table S3, Fig. S4B). The integration of the two replicate JUN ChIP-experiments shown here and in Fig. 1C revealed that more than 78% of the JUN peaks shown in Fig. 4 overlapped (Fig. S4C). The GO terms associated with genes co-bound by FOS and JUN from both replicates produced an almost identical list of terms and *P* values (Fig. S4D).

Approximately 40% of JUN peaks overlapped with FOS-binding sites, whereas 25% of FOS peaks overlapped with JUN, suggesting that JUN and FOS interacted with different partners at unique sites. In total, we identified 1227 genomic sites that were co-bound by FOS and JUN (Fig. 4C). Next, we annotated the closest genes to FOS and JUN peaks, intersected both gene populations and identified 910 genes that were bound by JUN only, 2190 bound by FOS only and 1388 genes that were annotated to both FOS and JUN peaks (Fig. 4D, Table S4). To examine whether the different binding configurations of JUN and FOS regulate different sets of genes, we further dissected these 1388 genes into those where FOS and JUN bound at identical sites (FOS:JUN co-bound, 1042 genes) and those where FOS and JUN bound at different sites (FOS and JUN distinct, 346 genes) (Fig. 4E, Table S4). Exemplary genome browser screen shots for these different patterns are shown in Fig. 4F. Gene ontology analysis revealed that FOS alone and JUN alone bound to genes associated with phosphorylation, metabolism and signalling (Fig. 4G). Interestingly, in contrast to these more general GO terms, FOS and JUN bound to heart- and muscle-linked genes; moreover, FOS:JUN dimers highly significantly co-bound vascular genes (Fig. 4G, lower right panel), suggesting a direct regulation of the vascular rather than the hematopoietic cell fate.

We next integrated FOS- and JUN-binding patterns with transcriptional changes ±DOX for day 1 blast culture, i.e. the time point of FOS and JUN ChIP-seq, by performing gene set enrichment analyses (GSEA). We found a high correlation between FOS or JUN binding and downregulated gene expression upon dnFOS induction (Fig. S5A), showing that both factors have largely activating roles. Next, we investigated the general pattern of expression of genes annotated to JUN only, FOS:JUN and FOS only binding events (Fig. S5B, left panel) all of which generally showed lower expression after 1 day of dnFOS induction than in control cells. This effect was strongest for FOS:JUN co-bound genes. Focusing on transcriptional changes of AP-1 target genes occurring during transitions of distinct developmental stages in the absence of DOX, we observed that AP-1 target genes were generally upregulated from FLK1^+^ to SM and from FLK1^+^ to HE1 cells, followed by a downregulation from HE1 to HE2 cells, but did not further change expression during the transition from HE2 to HP cells (Fig. S5B, right panels). Together with the GO analysis (Fig. 4G), this finding suggests that AP-1 is involved in a transient induction of vascular (endothelial and smooth muscle-related) genes in HB-derived cells, which are then downregulated during the EHT.

**Fig. 5.**
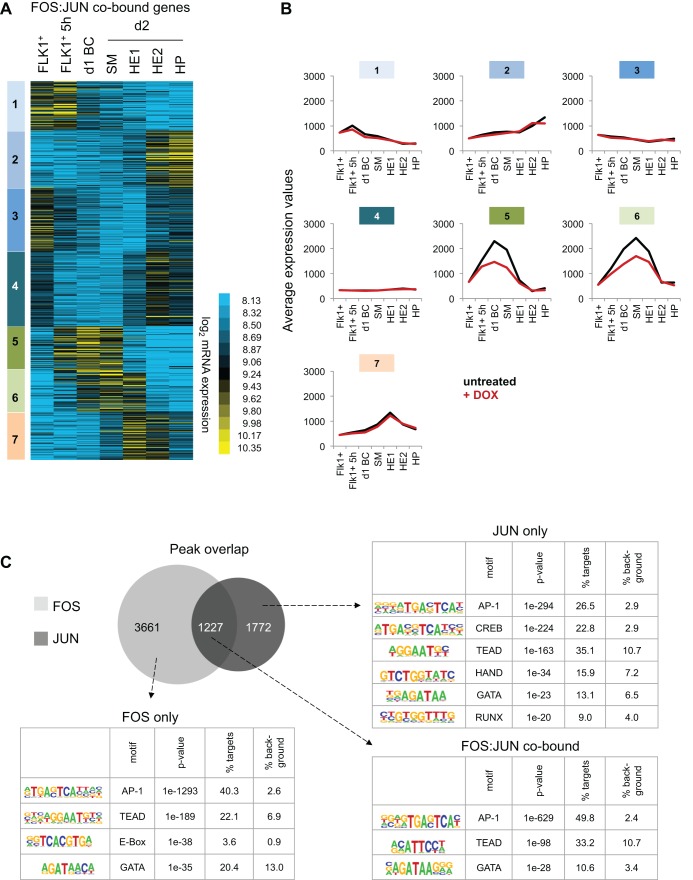
**AP-1 binding is required in SM and HE1 for the transient activation of genes, and is enriched at AP-1 and TEAD motifs.** (A) K-means clustering of log_2_ expression levels of genes associated with FOS:JUN co-bound sites. Each of the clusters (1-7) represents a specific pattern of gene expression levels. (B) Average mRNA microarray expression values of genes from clusters 1-7 as defined in B, without (black) and with (red) DOX for the different cell types of the differentiation. (C) Enriched transcription factor binding motifs in FOS-only, FOS:JUN and JUN-only peaks using HOMER *de novo* motif discovery analysis.

We next analysed the dynamic activity of all FOS:JUN co-bound genes in more detail by conducting k-means clustering of expression patterns in control FLK1^+^ cells, FLK1^+^ cells after 5 h and 1 day of BC, and in day 2 SM, HE1, HE2 and HP cells (Fig. 5A, Table S5). This analysis revealed seven gene clusters each with similar expression patterns during the differentiation of HB to HP cells. We then compared the averaged expression levels of each of the seven clusters during unperturbed differentiation with averaged expression levels of dnFOS-induced cells (Fig. 5B). Specific differences of expression levels between induced and control cells were apparent only in clusters 5 and 6, which contain 203 genes that are normally highly induced in day 1 blast culture, SM and HE1 cells. Sixty-eight of these genes were expressed at significantly lower levels when AP-1 was blocked (Table S6), among them we identified collagens (*Col1a1*, *Col4a1*, *Col5a1*) and *Bmp1* (the metalloprotease that supports collagen maturation by cleaving procollagens), vascular growth factors (*Vegfa*, *Pdgfc*, *Hbegf*, *Ctgf*), signalling ligands (*Bmp2*, *Tgfb*), genes encoding integrin ITGA11 and laminin LAMC2, and the transcription factor WT1, which was recently shown to be involved in angiogenesis (Duim et al., 2015; Katuri et al., 2014). These findings strongly suggest that during HB cell differentiation, AP-1 and in particular the FOS:JUN complex, activates a specific set of genes in SM and HE1 cells that are closely linked to and important for blood vessel formation. In the subsequent transition of HE1 cells to HE2 cells, i.e. cells that are fully committed to blood, these genes are downregulated and remain low in HP cells.

### AP-1 is required for *de novo* binding of the Hippo-signalling regulator TEAD4 at specific binding sites

The developmental stage-specific response to AP-1 inhibition indicated that these factors interact with cell type-specific sets of cis-regulatory elements. In order to identify additional TFs binding to these elements, we performed a *de novo* motif discovery analysis of FOS only, JUN only and FOS:JUN co-bound genomic sequences. For each of the three groups, the AP-1 consensus sequence was retrieved as top hit as expected (Fig. 5C, Fig. S5C). The CREB motif was also highly enriched and specific to JUN only peaks, reflecting the ability of JUN, but generally not FOS, to dimerize with ATF proteins (Fig. 5C, Fig. S5C) (Hai and Curran, 1991; Karin et al., 1997; van Dam and Castellazzi, 2001). A striking result from our motif analysis was the co-association of TEAD motifs within all FOS and JUN peaks (Fig. 5C). Moreover, in a motif co-occurrence clustering analysis computing enrichment against background colocalization frequencies, we found a close colocalization of AP-1 and TEAD motifs in FOS- and JUN-binding sites (Fig. S5D). The TEAD family of transcription factors contains four members, TEAD1-TEAD4, all of which share the same consensus sequence and are the downstream effectors of Hippo signalling (Meng et al., 2016). In the absence of Hippo signalling TEAD factors bind to DNA, but can activate gene expression only together with co-activators such as YAP, VGLL or p160 (Pobbati and Hong, 2013). Active Hippo signalling leads to phosphorylation of LATS1/2 by MST1/2, and to subsequent phosphorylation of YAP. Consequently, phospho-YAP is restricted to the cytoplasm and fails to activate TEAD-bound genes.

**Fig. 6.**
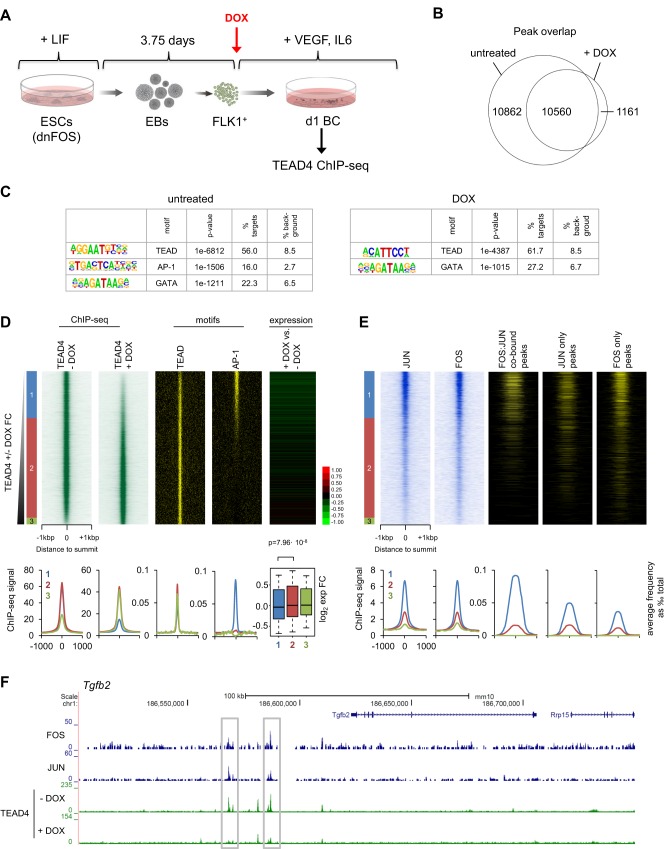
**AP-1 is required for *de novo* TEAD4 binding during differentiation of HB to HE.** (A) Experimental setup: dnFOS ESCs were differentiated into EBs FLK1^+^ cells and purified and cultured for 24 h under blast culture conditions with and without 1 µg/ml DOX. Cells of complete cultures were double crosslinked and chromatin was used for TEAD4 ChIP followed by genome-wide sequencing. (B) Overlap of TEAD4 ChIP-seq peaks in DOX- treated and untreated day 1 BC cells. (C) Enriched transcription factor-binding motifs within TEAD4 ChIP-seq peaks from DOX-treated and untreated samples using HOMER *de novo* motif discovery analysis. (D) TEAD4 ChIP-seq signal from DOX-treated and untreated cells (left), TEAD and AP1 motif presence (middle) and day 1 BC DOX/untreated gene expression fold change (right) ordered by increasing DOX/untreated TEAD4 ChIP-seq signal. Classes of peaks are indicated on the left and defined using cut-offs of ±1 log_2_ fold change, with class-specific average profiles as well as a boxplot showing gene expression fold change at the bottom. (E) JUN and FOS signals (left) and the presence of FOS:JUN intersecting peaks, and JUN- and FOS-only peaks (right) ordered as in D. Bottom: average profiles for peak presence for classes defined in (D). (F) Representative genome browser screenshot of the *Lamc2* locus showing TEAD4 loss at AP-1 binding sites following dnFOS induction.

Recent reports described the genomic colocalization of AP-1 and TEAD proteins in cancer cell lines (Diepenbruck et al., 2014; Liu et al., 2016; Verfaillie et al., 2015; Zanconato et al., 2015); however, how these two factors cooperate at specific genes was not studied. Thus, we investigated TEAD4 occupancy upon blocking AP-1 DNA binding by performing ChIP-seq in day 1 blast culture cells±dnFOS (Fig. S1E, Fig. 6A). For untreated cells, we obtained a total of 21,422 TEAD4 peaks, while the number of peaks for DOX-treated cells was almost halved (11,721 peaks, Fig. 6B and Fig. S6A). The decrease in peak numbers did not affect the overall genomic distribution: most of TEAD4 binding was still detected in distal intergenic or intronic elements (Fig. S6A). *De novo* motif discovery analysis showed that, with or without AP-1 inhibition, the TEAD motif was the top hit (Fig. 6C). However, AP-1 motifs in the DOX-treated sample were undetectable, indicating that, upon dnFOS expression, TEAD4 was no longer binding to sites containing AP-1 motifs (Fig. 6C, right panel). To substantiate this finding, we compared all TEAD4 peaks derived from treated and untreated cells, and ranked them according to the fold change of ChIP-seq signal intensity (Fig. 6D), which revealed three classes of TEAD4-binding sites (Table S7): class 1, comprising peaks that were specific to untreated cells and lost upon AP-1 inhibition (6787 sites, 30.6%); class 2, representing a shared class of TEAD4 binding that was not affected by dnFOS induction (14,404 sites, 65.1%); and class 3, containing a relatively small number of TEAD4 peaks that were gained (950 sites, 4.3%). The TEAD motif was found throughout all three classes, whereas the AP-1 motif was specific to class 1 peaks (Fig. 6D shows the motif heatmap and average profiles). When annotating the closest genes and analysing their expression fold change±DOX in day 1 blast culture, we found significantly lower expression only in class 1 genes, showing that the interaction between TEAD4 and AP-1 is required for high-level expression (Fig. 6D, right panel and box plot).

To investigate how TEAD4 binding was associated with JUN/FOS binding, we compared the ChIP-seq signals for FOS and JUN, as well as the previously identified FOS:JUN co-bound, JUN-only and FOS-only peaks using the same ranking of TEAD4 peaks (Fig. 6E). The majority of FOS- and JUN-binding events was contained in class 1 peaks, particularly FOS:JUN co-bound sites overlapped strongly with TEAD4 sites that were lost upon AP-1 inhibition. Intersecting the 203 FOS:JUN co-bound genes from cluster 5 and 6 (Fig. 5B) with class 1 TEAD4-bound genes, revealed that 175 of them, i.e. 86%, showed loss of TEAD4 binding upon dnFOS expression. Exemplary genome browser screen shots of some of these gene loci are shown in Fig. 6F and Fig. S6B. We further narrowed this gene population down to a total of 64 genes sharing the following features: transiently expressed during HB differentiation, co-bound by FOS:JUN, AP-1 dependently bound by TEAD4 and expressed at significantly lower levels upon dnFOS induction (Table S6). A high proportion of FOS and JUN peaks overlapped with TEAD4 binding without DOX (Fig. S6C). We found that the average TEAD4 ChIP-seq signal centred on such FOS or JUN peaks was reduced by more than twofold (Fig. S6D) upon AP-1 inhibition, again suggesting that a defined class of TEAD4-binding sites was AP-1 dependent.

In order to understand the dynamics of TEAD4 binding in development, we analysed TEAD4 ChIP-seq data in FLK1^+^ HB cells that we had published recently (Goode et al., 2016) and in addition generated a TEAD4 ChIP-seq dataset for purified HE1 cells (KIT^+^Tie2^+^CD41^−^), 75% of which overlapped with that of day 1 BC (Fig. S6A,E,G,H). In freshly purified FLK1^+^ HB cells, TEAD4 binding was already established at class 2 sites, but not at class 1 sites (Fig. S6E,G), which were specific for the HE stage. In summary, these analyses reveal that during the differentiation of HB cells into the HE cells, TEAD4 *de novo* binding occurs at cis-regulatory elements associated with a subset of vascular genes and that this binding is dependent on AP-1, suggesting a recruiting role for AP-1.

### AP-1- and TEAD-bound regions colocalize with occupied binding motifs for multiple inducible transcription factors in open chromatin of hemogenic endothelium cells but not thereafter

**Fig. 7.**
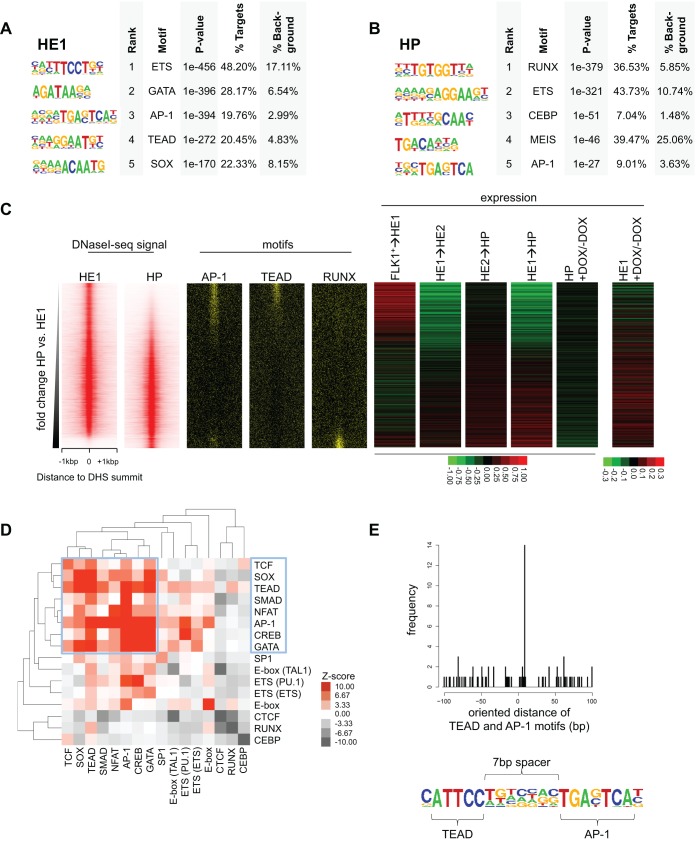
**AP-1 and TEAD footprints occur together in HE1 DHS peaks and are strongly reduced in HP hypersensitive sites.** (A) Transcription factor-binding motifs enriched in HE1-specific DHSs employing HOMER *de novo* motif discovery. (B) As in A, examining HP-specific DHSs. (C) Heatmaps depicting HE1 and HP DNaseI-seq signal (left), motif (middle) and gene expression fold change (right) sorted based on increasing HP/HE1 fold change. (D) Co-occurrence clustering of factor binding motifs specifically occupied (footprinted) in HE1 demonstrating the co-occurrence of motifs for signalling-responsive transcription factors. Z-scores represent enrichment over 1000 equally sized, random sub-samplings in HP DHSs. (E) AP-1 and TEAD motifs are arranged in a specific conformation. Distribution of AP-1 motif start distances to TEAD motif starts, aligned by TEAD motif orientation (top). Bottom: composite TEAD-AP1 motif with 7 bp spacer.

We have recently shown that the interaction between YAP and TEAD factors is required for the differentiation of hematopoietic cells from FLK1^+^ cells purified from ESCs and mouse embryos but not in HP cells. This was accompanied by an activation of Hippo signalling, leading to the absence of TEAD and YAP in the nucleus of HP cells (Goode et al., 2016). The analysis presented here shows that on average all genes bound by TEAD4 showed similar expression patterns during HB differentiation: a strong induction from FLK1^+^ to SM and from FLK1^+^ to HE1 cells (Fig. S6F). GO analysis showed that all three classes of TEAD4 sites were associated with genes involved in cell adhesion and vasculogenesis (Fig. S6I). However, all of these genes were strongly downregulated from HE1 to HE2 cells, and expression did not change in HP cells, suggesting that AP-1 cooperates with TEAD prior to, but not after, the EHT. We tested this idea by performing deep DNaseI sequencing in FACS-purified HE1 (KIT^+^TIE2^+^CD41^−^) and HP (KIT^+^TIE2^−^CD41^+^) cells. We obtained 68,691 DNaseI hypersensitive sites (DHSs) for HE1 cells and 63,712 DHSs for HP cells [44,216 of them were shared between the two samples (Fig. S7A,B)]. *De novo* motif discovery analysis revealed that ETS, GATA, AP-1, TEAD and SOX motifs were enriched in HE1 open chromatin regions, whereas motifs for the hematopoietic transcription factors RUNX, ETS, CEBP and MEIS, and also AP-1 motifs, were found in HP-specific DHSs (Fig. 7A,B). Upon ranking of the fold change of the union of HE1 and HP DHSs, we identified specific patterns of motif distribution: TEAD, SOX and GATA motifs were distinct from HE1-specific DHSs, whereas RUNX and CEBP motifs were characteristic of HP-unique DHSs (Fig. 7C, Fig. S7C). The AP-1 motif was found in both HE1-specific and HP-specific DHSs, but not at shared sites, again highlighting the different function of these binding sites in endothelial and hematopoietic cells. When plotting FOS and JUN ChIP-seq signal of day 1 blast culture cells using the same ranking, the highest signal was obtained in HE1-specific DHSs (Fig. S7C), concordant with binding of AP-1 in HE1 cells. We then annotated these binding sites to the promoters of the closest genes, calculated fold changes in expression for developmental transitions and plotted the resulting heatmaps in the same ranking (Fig. 7C, right panel). Generally, genes annotated to HE1-specific DHSs were upregulated from FLK1^+^ to HE1 and downregulated from HE1 to HE2, whereas their expression did not change from HE2 to HP. By contrast, genes annotated to HP-specific sites showed a gradual upregulation throughout all transitions from HB to HP. Furthermore, HP-specific sites were associated with AP-1, RUNX and CEBP motifs, as seen with FOS binding in mast cells (Calero-Nieto et al., 2014) and with reduced transcription in HP cells upon dnFOS induction (Fig. 7C, Fig. S7C). These observations support our hypothesis of AP-1 playing a role during the differentiation of HP cells to myelomonocytic cells. However, TEAD appears to be involved only in a HE1-specific context.

To examine the interaction between TEAD4 and AP-1 at base-pair resolution, we performed digital footprinting analysis on our DNaseI-seq data using the Wellington algorithm (Piper et al., 2013), and identified 70,522 and 53,964 regions in HE1 and HP cells that were protected from DNaseI digestion, respectively (Fig. S7D). Interestingly, the overlap between those was found to be not significant (*P*=1), denoting a markedly different repertoire of occupied TF-binding sites between the two stages. Both AP-1 and TEAD motif-containing footprints were enriched in HE1 cells, and occupancy of these motifs in HE1 cells was strongly reduced in HP cells (Fig. S7E-H). Moreover, when centred on FOS and JUN ChIP-seq peaks of day 1 blast culture, AP-1 motifs were preferentially occupied in HE1 cells rather than HP cells (Fig. S7I), and were associated with class 1 genomic sites (see Fig. 6D, AP-1-dependent TEAD4 sites) for HE1 but not for HP (Fig. S7J). This assay also allowed us to investigate the co-occupancy of other transcription factor binding motifs with AP-1 and TEAD by clustering HE1-footprinted motifs using bootstrapping analysis, which determines the significance of co-clustering motifs within 50 bp of DNA. We found a significant co-occurrence of occupied AP-1 and TEAD motifs together with occupied motifs for other signalling-inducible transcription factors such as SMAD, NFAT, CREB and TCF, together with motifs for tissue-specific factors such as SOX and GATA (Fig. 7D). Our results are supported by previous reports describing a co-association of GATA and AP-1 in mature vascular cells (Linnemann et al., 2011). By contrast, in HP cells, occupied motifs for the hematopoietic transcription factors ETS, E-Box (TAL1), RUNX- and CEBP predominantly clustered together (Fig. S7K). Importantly, at co-occurring AP-1 and TEAD footprinted motifs in HE1 cells, we found a bias towards a defined oriented distance between both motifs with a 7 bp spacing (Fig. 7E, Fig. S7L). This composite motif likely indicates direct interaction between AP-1 and TEAD, as was identified from other composite motifs previously (Chen et al., 1998; Cockerill et al., 1995; Hollenhorst et al., 2009). In conclusion, these analyses show that AP-1 and TEAD co-occupy a subset of genomic sites that are accessible in HE1 cells but inaccessible in HP cells. Genes associated to these elements are transiently activated from HB to HE1 commitment but downregulated in HE2 and HP cells.

In summary, we propose a model in which AP-1 (FOS:JUN) promotes the differentiation of vascular smooth muscle cells and also binds to vascular genes in the hemogenic endothelium (Fig. S8). During the transition from HB to HE1 cells, AP-1 is needed for the transient induction of such genes, which are subsequently downregulated when cells further differentiate to blood-committed cells. A substantial proportion of these genomic sites is co-occupied by the Hippo signalling-related TF TEAD4 and we show that AP-1 is required for its *de novo* binding to these elements. Although AP-1 is also important later for the maturation of HP cells, it no longer co-localizes with TEAD4 at this developmental stage. Altogether, our study highlights the versatility of the ESC differentiation system to dissect the molecular mechanism of specific knockout phenotypes that would otherwise not be amenable to biochemical studies.

## DISCUSSION

### Inhibition of AP-1 activity shifts the balance between vascular and hematopoietic cell fates

By identifying and studying the function of genomic regions bound by signalling-responsive TFs we can obtain a first mechanistic insight into how such processes are coordinated and which signalling processes are involved. Here, we have used such a bottom-up approach to study the function of the AP-1 transcription factor family in hematopoietic specification. Our results uncover a role for AP-1 in the establishment of the smooth muscle and vascular program from hemangioblast cells, identify the genes involved in this process and pinpoint the developmental stage at which this occurs. Lineage-tracing experiments demonstrated that SM cells and HE cells develop independently of each other from HB cells (Stefanska et al., 2014). We hypothesize that the impaired induction of the vascular gene expression program in the hemogenic endothelium after AP-1 inhibition leads to an imbalanced cell fate decision towards blood-committed cells and consequently to more HP cells. This is consistent with a previous report, stating that repression of arterial/vascular genes in HE is sufficient for hematopoietic fate acquisition (Lizama et al., 2015). *Runx1* expression is not affected by dnFOS expression (Table S1) and thus hematopoietic progenitor cells undergo the EHT and upregulate the RUNX1-dependent CD41 marker (Lie-A-Ling et al., 2014). In the presence of DOX, the cells cannot mature to CD45^+^ and CD11b^+^ cells, indicating that further differentiation is blocked. However, in the absence of dnFOS they resume their normal differentiation behaviour and form different types of colonies, indicating that this block is reversible.

In addition to changes in the differentiation pattern, we observed enhanced proliferation throughout ESC differentiation in the presence of dnFOS. *Cdkn2a* and *Cdkn2b*, which encode the negative cell cycle regulators p16INK4a and p15INK4B, respectively, were bound by AP-1 and expressed at lower levels after AP-1 inhibition. Both *Cdkn2a* and *Cdkn2b* are expressed at very high levels in day 1 BC and SM cells under normal conditions (Fig. 3); a lower expression of these genes upon AP-1 inhibition would preferentially enhance the proliferation of SM cells compared with HE or HP cells. However, we found that the relative proportion of HE2 cells and not SM cells was increased. Furthermore, after only 1 day of blast culture, when cell numbers ±DOX were identical, we already observed reduced proportions of SM cells. In addition, our gene expression analyses using purified cells show clearly that the shift in balance between vascular and hemogenic fate is based on true alterations in gene expression and not just a change in proliferation.

Among the genes that require AP-1 for their induction, we identified TFs, ligands, membrane receptors, cytoskeleton and extracellular matrix proteins (Table S6). However, our data also show that AP-1 is not a cell fate-deciding factor, but plays a role in modulating cell fate decisions, most likely in concert with true master regulators such as RUNX1. We suggest that signalling-responsive TFs boost the expression of genes that actually define a cell, such as metabolic genes, extracellular matrix and focal adhesion genes, and other effector genes. One of these genes encodes the tyrosine kinase AXL (O'Bryan et al., 1991). AXL has been shown to be involved in vasculogenesis, both together with its ligand GAS6 and also ligand independently through VEGFA-VEGFR2 crosstalk and subsequent PI3K activation (Melaragno et al., 1999; Ruan and Kazlauskas, 2012). Thus, reduced *Axl* expression leads to a compromised response to VEGFA and in return to attenuated endothelial function. Another gene we have identified as being AP-1 dependent encodes the transcription factor Wilms' tumour 1 (WT1) (Call et al., 1990; Gessler et al., 1990). This zinc-finger protein is known to be essential for blood vessel formation, particularly for coronary vessels (Duim et al., 2015; Katuri et al., 2014).

### TEAD requires AP-1 to activate vascular genes in the hemogenic endothelium

We have previously shown that the interaction between TEAD and YAP is absolutely required for hematopoietic specification (Goode et al., 2016). An important finding of our study is therefore that AP-1-dependent transiently expressed vascular genes in the HE are bound by both AP-1 and TEAD4. During HB differentiation (day 1 BC) and in HE1 cells, TEAD4 requires AP-1 to be recruited to joint binding sites, indicating a crosstalk between AP-1 (MAPK and others) and Hippo signalling pathways. In HB cells, these regulatory elements are not yet bound by TEAD4, although TEAD4 binding is present at other genomic sites at this developmental stage. We were unable to ChIP JUN in FLK1^+^ cells (data not shown) as *Fos* is expressed at only low levels, but is then upregulated in the blast culture (Fig. S1F), suggesting that, in differentiating HB cells, AP-1 becomes active, recruits TEAD4 and activates gene expression. In HE2 cells, when a hematopoietic fate is acquired in response to the upregulation of *Runx1*, these genes are then repressed. Expression remains low in HP cells and AP-1 and TEAD footprints are no longer detectable. In a recent report, an early hemogenic precursor cell type in mammalian placentas was identified and its transcriptome was studied (Pereira et al., 2016); it was found that the genes specific for hemogenic precursors are linked to AP-1-, TEAD-, TCF- and GATA motifs, supporting the findings of our digital footprinting experiments, which provides direct evidence that these sites are occupied.

### AP-1- and TEAD4-binding cis-regulatory elements form signalling hubs in the hemogenic endothelium

Only recently have insights been gained into the role of signalling pathways involved in embryonic blood specification. For example, pro-inflammatory cytokines including TNFα and IFNγ were reported to promote HSC emergence by acting either upstream or downstream of Notch (Espín-Palazón et al., 2014; He et al., 2015; Li et al., 2014; Sawamiphak et al., 2014). WNT signalling via β-catenin is essential for the generation of the hemogenic endothelium (Ruiz-Herguido et al., 2012). Catecholamines from the sympathetic nervous system are important components of the developing HSC microenvironment (Fitch et al., 2012) and BMP4 signalling leads to a SMAD1/5-mediated repression of *Erk* transcription in HE cells, implying a role for mitogen-activated protein kinase (MAPK) signalling in these cells (Zhang et al., 2014). The specification of HSCs from the hemogenic endothelium is a tightly regulated biological process that is controlled by dynamic changes of signalling and TF activity. The HE is a heterogeneous tissue in which only a fraction of cells commits to blood while others remain part of the endothelial layer and may be important for providing signals to the microenvironment to support the EHT (Thambyrajah et al., 2016). Our digital footprinting analyses suggest that HE-specific occupied binding sites form signalling hubs that bind TFs responding to different signalling pathways, all of which impact on hematopoietic specification (Kim et al., 2014) and which are becoming decommissioned after the EHT. We propose a model in which various stimuli regulate the expression of the same set of genes but via different factors: WNT signalling via TCFs, Hippo signalling through TEADs, TGFβ and BMP signalling through SMADs, calcium signalling through NFAT and MAPK/cyclic AMP signalling through AP-1, ATFs and CREB. It remains unknown, however, whether all of these signals are active simultaneously in one cell or if subpopulations of HE cells respond to individual signals at a given time point.

In conclusion, our bottom-up strategy identifies subtle and varied roles for AP-1 transcription factor family members during embryonic blood development. Future work will determine how different signalling pathways regulate the transcriptional activity of genes associated with signalling hubs.

## MATERIALS AND METHODS

### Construction of p2lox-dnFOS plasmid, ESC transfection, culture and differentiation

The Flag-tagged dominant-negative FOS (dnFOS) construct was PCR amplified from CMV plasmid [CMV500-8584hep-fosLZ(MO)], A-FOS [kindly provided by Charles Vinson, National Cancer Institute, Bethesda, USA (Olive et al., 1997)], and *Hin*dIII and *Not*I sites were introduced. The GFP of the p2lox plasmid [kindly provided by Michael Kyba, Lillehei Heart Institute, University of Minnesota, USA (Kyba et al., 2002)] was exchanged for Flag-tagged dnFOS.

A2lox ESCs [a gift from Michael Kyba (Kyba et al., 2002)] or Bry-GFP ESCs were cultured and transfected as described previously (Gilmour et al., 2014; Lichtinger et al., 2012; Regha et al., 2015). ESCs were differentiated as described previously (Gilmour et al., 2014; Lancrin et al., 2010; Lichtinger et al., 2012). Further details can be found in the supplementary Materials and Methods.

### Methylcellulose hematopoietic progenitor colony assay

Floating progenitors were harvested from the supernatant of day 3 blast cultures ±DOX, counted and plated in triplicates at 10,000 cells per ml of MethoCult methylcellulose (Stem Cell Technologies, M3434) without DOX. After 7-10 days, colonies were counted and classified as BFU-E, CFU-Mix or CFU-GM.

### Giemsa staining

Individual colonies of methylcellulose cultures were picked, washed in PBS and immobilized on microscope slides by cytospin (4 min, 800 rpm, Shandon Cytospin 3). Cells were fixed with methanol for 1 min, air-dried, Giemsa-stained for 3 min (Sigma, GS500) and analysed by microscopy.

### Flow cytometry and FACS sorting

Cells at the developmental stages indicated were harvested and stained, and DNA content was measured. Further details can be found in the supplementary Materials and Methods

### RNA extraction, RT-qPCR and gene expression microarray analyses

For RNA extraction, cell pellets were re-suspended in Trizol (Invitrogen) and purified according to the manufacturer's protocol. The microarrays used were Agilent SurePrint G3 Mouse 8X60K microarrays (catalogue number G4852A-028005).

cDNA was prepared from the mRNAs using MMLV-RT (Promega M170A) and oligo dT primers according to the manufacturer's recommendations. Real-time PCR was performed with SYBR Green PCR master mix (Life Technologies, 4309155) and in an ABI Stepone real-time PCR machine. Further details and primer sequences can be found the supplementary Materials and Methods.

### Immunofluorescence staining

dnFOS ESCs were cultured for 2 days on glass cover slips in 24-well plates with MEFs and ESC medium, treated and incubated with primary and secondary antibodies. Further details can be found in the supplementary Materials and Methods.

### Western blotting

Protein extracts in Laemmli buffer were separated on 4-20% pre-cast gradient SDS-PAGE gels (Biorad) and western blots prepared by wet transfer onto nitrocellulose membrane. Further details can be found in the supplementary Materials and Methods.

### Chromatin immunoprecipitation (ChIP) and library preparation

Cells (2×10^6^ to 5×10^6^) were harvested and washed in PBS before a two-step crosslinking procedure. First, proteins were crosslinked by incubating cells for 45 min at room temperature in PBS supplemented with 0.83 mg/ml Di(N-succinimidyl) glutarate (DSG, Sigma 80424). After three PBS washes, formaldehyde crosslinking of proteins and DNA was carried out for 10 min at room temperature at a concentration of 1% formaldehyde (Pierce) in PBS. Formaldehyde was quenched by adding glycine to a final concentration of 100 mM and crosslinked cells were washed twice in ice-cold PBS. Nuclei isolation, sonication and ChIP were performed as previously described (Gilmour et al., 2014; Lichtinger et al., 2012; Regha et al., 2015). Further details and ChIP primer sequences can be found in the supplementary Materials and Methods.

### DNaseI digestion and library preparation

DNaseI digestion and libraries were prepared as described previously (Ptasinska et al., 2012) and deep sequencing was carried out on Illumina HiSeq2000.

### Luciferase assay

RAW cells were grown in DMEM medium supplemented with 10% FCS, penicillin and streptomycin, and plasmids were transfected using Invitrogen Lipofectamine according to the manufacturer's guidelines. Samples were prepared using Promega DnaI Luciferase Reporter Assay. Further details can be found in the supplementary Materials and Methods.

### Data accessibility, processing and analysis

#### High-throughput sequencing processing, peak detection and generation of coverage tracks

Briefly, alignment of reads was performed using bowtie; peak detection and coverage track generation with macs14. Further details can be found in the supplementary Materials and Methods.

#### ChIP-seq and DNaseI-seq rankings, heatmaps and average profiles

Briefly, ChIP-seq, DNaseI-seq heatmaps and average profiles were derived from ranked regions using tag count retrieved ±1000bp around summits via HOMER. Further details can be found in the supplementary Materials and Methods.

#### Motif discovery, heatmaps and average profiles

In brief, motif discovery, heatmaps and average profiles were carried out using HOMER. Further details can be found in the supplementary Materials and Methods.

#### Gene ontology analyses

Gene ontology (GO) analyses (biological process and KEGG pathway ontology classes) were performed using DAVID (Huang da et al., 2009) for all figures except Fig. 4.

#### Venn diagrams

Venn diagrams using gene names were derived using BioVenn (Hulsen et al., 2008). For high-throughput sequencing peaks, the makeVennDiagram function of the ChIPpeakAnno R package (Zhu et al., 2010) was used, which was also used to compute hypergeometric p-values of intersections. Further details can be found in the supplementary Materials and Methods.

#### Digital genomic footprinting

Digital genomic footprinting was performed using Wellington (Piper et al., 2013) using standard parameters. Further details can be found in the supplementary Materials and Methods.

#### Motif co-occurrence clustering

Essentially, motif co-occurrence clustering was performed on enrichments of co-occurring footprinted motifs over a random background, using cluster 3.0. Further details can be found in the supplementary Materials and Methods.

#### Gene set enrichment analyses

Gene-set enrichment analyses were performed with the GSEA analysis suite (Subramanian et al., 2005). Further details can be found in the supplementary Materials and Methods.

#### K-means clustering

Expression values of the closest gene were recovered for FOS:JUN co-bound peaks. K-means clustering was performed aiming for seven gene glusters using cluster 3.0 using -g 2 -k 7 -na -ng as parameters.

#### Motif distances

In summary, distributions of distances between the TEAD motif end and AP-1 motif start coordinates were computed and plotted using HOMER and R. Further details can be found in the supplementary Materials and Methods.

#### Microarray data analysis

Microarray data analysis was performed as previously described (Lichtinger et al., 2012), using the limma R package. Further details can be found in the supplementary Materials and Methods.

### Public datasets

The HB DNaseI-seq and TEAD4 ChIP-seq (Goode et al., 2016), as well as mast cell FOS ChIP-seq (Calero-Nieto et al., 2014) datasets were downloaded as SRA archives from the Gene Expression Omnibus accession numbers GSM1692782, GSM1968747 and GSM1167585, respectively. These were converted to fastq via sra-toolkit 2.5.2 (Leinonen et al., 2011). These datasets were processed in the same way as other high-throughput sequencing samples from this study.
